# A Preliminary Study of the Role of Endothelial-Mesenchymal Transitory Factor SOX 2 and CD147 in the Microvascularization of Oral Squamous Cell Carcinoma

**DOI:** 10.7759/cureus.52265

**Published:** 2024-01-14

**Authors:** Vasileios Zisis, Pinelopi A Anastasiadou, Athanasios Poulopoulos, Konstantinos Vahtsevanos, Konstantinos Paraskevopoulos, Dimitrios Andreadis

**Affiliations:** 1 Oral Medicine and Pathology, Aristotle University of Thessaloniki, Thessaloniki, GRC; 2 Oral and Maxillofacial Surgery, Papanikolaou Hospital, Aristotle University of Thessaloniki, Thessaloniki, GRC

**Keywords:** immunohistochemistry staining, cd147, sox 2, oral leukoplakia, oral cancers, cancer stem cells

## Abstract

Introduction: The aim of this study was to detect the possible endothelial expression of embryonic-type cancer stem cells (CSC) marker SOX2 and the stemness-type CSC marker CD147 in oral potential malignant disorders (OPMDs), oral leukoplakia (OL) in particular, and oral squamous cell carcinoma (OSCC).

Methods: This study focuses on the immunohistochemical pattern of expression of CSC protein-biomarkers SOX2 and CD147 in paraffin-embedded samples of 21 OSCCs of different grades of differentiation and 30 cases of OLs with different grades of dysplasia, compared to normal oral mucosa.

Results: The protein biomarker SOX2 was expressed in the endothelial cells, but without establishing any statistically significant correlation among OSCC, OL, and normal tissue specimens. However, SOX endothelial staining was noticed in 7/30 (23.3%) cases of OL (one non-dysplastic, one mildly dysplastic, one moderately dysplastic, and four severely dysplastic cases) and 5/21 (23.8%) cases of OSCC (two well-differentiated, one moderately differentiated, and two poorly differentiated cases). Although CD147 is expressed in normal oral epithelium, OL, and OSCC neoplastic cells, its vascular-endothelial expression was noticed in only 2/5 (40%) cases of normal oral epithelium, 1/30 (3.3%) cases of OL (one severely dysplastic case), and 4/21 (19%) cases of OSCC (two well-differentiated, one moderately differentiated, and one poorly differentiated case). Therefore, no statistically significant correlation among OSCC, OL, and normal tissue specimens was established.

Conclusion: The endothelial presence of SOX2 both in oral potentially malignant and malignant lesions suggests that SOX2 may be implicated in the microvascularization process and associated with the degree of dysplasia in OL. The expression of CD147 may be attributed both to local inflammation and tumorigenesis. The implementation of CD147 in larger groups of tissue samples will shed some light on its role in cancer and inflammation. The evidence so far supports the need for more studies, which may support the clinical significance of these novel cancer stem cell biomarkers.

## Introduction

The protein biomarker SOX2 is found in embryonic cancer stem cells (CSCs), with well-established implications for oral potential malignant disorders (OPMDs) and oral squamous cell carcinoma (OSCC) [1]. In addition, it has been considered a marker of vascular expression, indicating its participation in endothelial proliferation [2] and possibly neo-vascularization [3], a critical process for oral tumorigenesis [4]. The differentiation of endothelial cells (ECs) is crucial for facilitating the vascularization of tissues and ensuring the maintenance of vascular homeostasis [2]. In conjunction with the process of tissue development, ECs originate from pluripotent precursor cells, which differentiate into the endothelial lineage [5,6]. In terms of local inflammation, the quiescent endothelium turns into an active endothelium [7]. Cancer may exhibit characteristics reminiscent of developmental processes, including the overexpression of morphogenic factors [8], the disruption of stem cell regulation [9], the aberrant formation of blood vessels [10], as well as the occurrence of cell differentiation in atypical locations [11]. Endothelial-mesenchymal transitions (EndMTs) are a typical occurrence in neural crest development, cardiac valves, and neovascularization [12-18].

The significance of the SOX transcription factors in vascular development has been well documented [2]. Vascular SOX transcription factors are a group of proteins that play a crucial role in regulating gene expression in vascular tissues [2]. The sex-determining region Y (SRY) gene, named so as it is located in the sex-determining region of the Y-chromosome, was initially identified as a gene responsible for determining the development of sexes in humans [19,20]. The SRY-related high mobility group (HMG) box of DNA-binding proteins is commonly known as SOX transcription factors. This family encompasses over 20 SOX genes [21]. The SOX transcription factors are distinguished by the HMG box, a 79-amino-acid DNA-binding motif. These motifs vary depending on their role in developmental programs [22,23].

The stemness-type CSC biomarker CD147 controls the tumor microenvironment and angiogenesis through paracrine signaling [24,25]. The biomarker CD147 is expressed in over 20 distinct cancer types, and its expression level is associated with an unfavorable prognosis, including decreased overall survival rates [26]. Furthermore, the excessive presence of CD147 is linked to decreased overall survival in patients with primary melanomas [27] and the spread of cancer to deeper layers of skin, indicating that CD147 serves as a significant predictive marker for the aggressiveness of melanoma [28]. A retrospective investigation found a link between the overexpression of CD147 and the presence of malignant characteristics, such as lymph node invasion and metastasis, in non-small-cell lung cancer [29]. The expression of the biomarker CD147 in cervical cancer has a role in regulating invasion and lymph node metastasis by affecting lipid metabolism, and in particular, CD147 promotes the growth of new lymphatic vessels in tumors through the synthesis of fatty acids [30]. The majority of tumor cells have been observed to exhibit high levels of CD147 on their cell membrane [31,32]. Biomarker CD147 is believed to engage in paracrine interactions with the tumor microenvironment, stimulating the activation of cells such as stromal fibroblasts. This activation leads to the transformation of fibroblasts into myofibroblasts, resulting in increased expression of matrix metalloproteinases (MMPs) and promoting the invasion of tumor cells [26,33]. Finally, CD147 has been demonstrated to engage with endothelial cells in a paracrine way, hence stimulating angiogenesis [34]. Biomarker CD147 enhances the development of new blood vessels through the control of VEGFR-2, thus leading to increased movement and tube formation in endothelial cells, as well as tumor growth in living organisms [24,25]. The aim of this study was to investigate the immunohistochemical pattern of endothelial expression of SOX2 and CD147 in vessels beneath the epithelium of the most common OPMD, oral leukoplakia (OL), and OSCC.

## Materials and methods

The paraffin-embedded tissue samples of normal oral epithelium, OL, and OSCC were derived from biopsies conducted between 2009 and 2019 in the Department of Oral Medicine and Pathology at the School of Dentistry, Oral and Maxillofacial Surgery Clinic of G.Papanikolaou General Hospital, Aristotle University, and the Oral and Maxillofacial Surgery Clinic of St. Luke Hospital, both located in Thessaloniki, Greece. The study was approved by the ethics committee of the School of Dentistry, Aristotle University (approval no. Nr 8/03.07.2019). The presence of adequate precancerous or cancerous tissue was the main inclusion criteria. The exclusion criteria included the lack of adequate tissue. The study is semiquantitative research and examines the immunohistochemical, vascular-endothelial pattern of expression of SOX2 and CD147 in tissue samples from 30 cases of OLs and 21 OSCCs in comparison to five cases of normal mucosa (a healthy epithelium adjacent to reactive benign lesions such as fibromas). The epidemiological and topographical data of the examined cases are summarized in Table 1.

**Table 1:** Summary of the epidemiological and topographical data of the examined cases OSCC: Oral squamous cell carcinoma

Case no.	Category	Location	Age (in years)	Gender
1	Leukoplakia	Tongue	44	Female
2	Leukoplakia	Tongue	60	Female
3	Leukoplakia	Tongue	58	Male
4	Leukoplakia	Tongue	67	Female
5	Leukoplakia	Tongue	62	Female
6	Leukoplakia	Cheek	66	Male
7	Leukoplakia	Cheek	67	Male
8	Leukoplakia	Tongue	43	Male
9	Leukoplakia	Buccogingival sulcus	75	Female
10	Leukoplakia	Tongue	50	Male
11	Leukoplakia	Buccogingival sulcus	59	Male
12	Leukoplakia	Tongue	75	Male
13	Leukoplakia	Tongue	64	Male
14	Leukoplakia	Tongue	45	Male
15	Leukoplakia	Palate	72	Male
16	Leukoplakia	Tongue	84	Female
17	Leukoplakia	Tongue	61	Female
18	Leukoplakia	Lip	38	Female
19	Leukoplakia	Tongue	46	Male
20	Leukoplakia	Gingiva	12	Female
21	Leukoplakia	Tongue	45	Female
22	Leukoplakia	Tongue	67	Male
23	Leukoplakia	Cheek	60	Female
24	Leukoplakia	Tongue	68	Female
25	Leukoplakia	Tongue	69	Male
26	Leukoplakia	Tongue	68	Female
27	Leukoplakia	Cheek	58	Female
28	Leukoplakia	Cheek	61	Female
29	Leukoplakia	Cheek	75	Male
30	Leukoplakia	Corner of the mouth	37	Male
31	OSCC	Tongue	47	Female
32	OSCC	Tongue	53	Female
33	OSCC	Cheek	45	Male
34	OSCC	Tongue	77	Female
35	OSCC	Cheek	66	Male
36	OSCC	Cheek	61	Male
37	OSCC	Mouthfloor	76	Female
38	OSCC	Tongue	75	Male
39	OSCC	Tongue	79	Female
40	OSCC	Cheek	78	Female
41	OSCC	Tongue	72	Female
42	OSCC	Tongue	52	Male
43	OSCC	Tongue	60	Male
44	OSCC	Tongue	77	Female
45	OSCC	Tongue	80	Female
46	OSCC	Mouthfloor	82	Female
47	OSCC	Lip	58	Male
48	OSCC	Lip	77	Male
49	OSCC	Tongue	73	Female
50	OSCC	Tongue	67	Female
51	OSCC	Tongue	43	Male
52	Normal	Tongue	49	Female
53	Normal	Cheek	81	Male
54	Normal	Cheek	59	Female
55	Normal	Tongue	69	Male
56	Normal	Tongue	72	Female

The protocol of the immunohistochemical technique included the use of an anti-SOX2 antibody (sc-365823, Santa Cruz Biotechnology, Dallas, TX, USA) and an anti-CD147 antibody (sc-21746, Santa Cruz Biotechnology). The evaluation of the staining of SOX 2 and CD147 was defined as positive or negative. In the presence of at least one positively stained endothelial cell, the section was deemed positive. Statistical analysis was performed using SPSS Statistics version 25.0 (IBM Corp., Armonk, NY, USA) with the Pearson chi-square test and the Fisher’s exact test depending on the sample size. The significance level was set at 0.05 (p=0.05).

## Results

Findings regarding SOX2 expression

Even though SOX2 is expressed in normal oral epithelium, OL epithelium, and OSCC neoplastic cells, its vascular-endothelial expression was not specific, and it was noticed only in 7/30 (23.3%) cases of OL (one non-dysplastic, one case of mild dysplasia, one of moderate dysplasia, and four cases of severe dysplasia) and 5/21 (23.8%) cases of OSCC (two well-differentiated, one moderately differentiated, and two poorly differentiated cases). In contrast, there was an absence of staining in normal mucosa endothelium (Table 2).

**Table 2:** The demographical and clinical manifestation details of the patients from whom positively stained tissue samples were derived. OSCC: Oral squamous cell carcinoma, OL: Oral leukoplakia

Case no.	Diagnosis	Location	Gender	Age (in years)
7	Moderately dysplastic OL	Cheek	Male	67
8	Severely dysplastic OL	Tongue	Male	43
9	Severely dysplastic OL	Buccogingival sulcus	Female	75
11	Severely dysplastic OL	Buccogingival sulcus	Male	59
13	Severely dysplastic OL	Tongue	Male	64
19	Mildly dysplastic OL	Tongue	Male	46
21	Non-dysplastic OL	Tongue	Female	45
32	OSCC/Moderately differentiated	Tongue	Female	53
34	OSCC/Poorly differentiated	Tongue	Female	77
44	OSCC/Poorly differentiated	Tongue	Female	77
47	OSCC/Well-differentiated	Lip	Male	58
48	OSCC/Well-differentiated	Lip	Male	77

**Figure 1:**
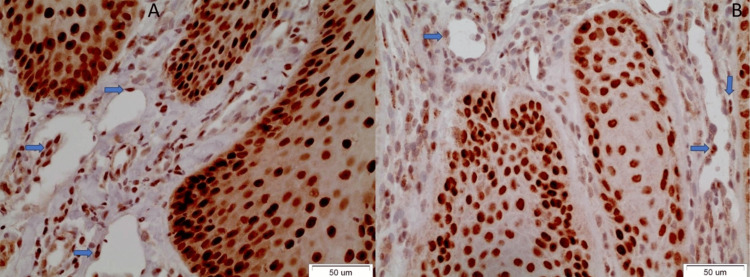
The blue arrows indicate the positively stained endothelial cells. A: Non-dysplastic OL (case no. 21), B: Severely dysplastic OL (case no. 8) OL: Oral leukoplakia

The staining was negative in all of the normal cases. The positive OL cases included one non-dysplastic OL, one mildly dysplastic OL, one moderately dysplastic OL, and four severely dysplastic OLs (Figure 1).

**Figure 2:**
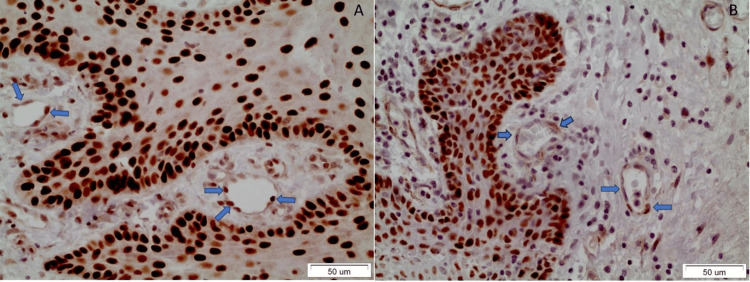
The blue arrows indicate the positively stained endothelial cells. A: Well-differentiated OSCC (case no. 47), B: Poorly differentiated OSCC (case no. 34) OSCC: Oral squamous cell carcinoma

Therefore, the positivity of endothelial cells increases as dysplasia progresses, since the majority of positive OL cases are severely dysplastic ones. In these cases, the positively stained endothelial cells of the vessels were adjacent to the superficial dysplastic epithelium (displaying cytoplasmic and membranous staining beneath the dysplastic OL epithelium). However, no statistically significant difference was noticed compared to the normal epithelium (Fisher’s exact test, p-value=0.999). Interestingly, this partial positivity in worse cases of OL was dramatically increased in the endothelial cells of 7/21 OSCC samples, although no statistical significance was noticed compared to OL samples (Fisher’s exact test, p-value=0.226). The pattern of positive expression in OSCC cases includes the cytoplasmic and membranous staining of endothelial cells adjacent to the cancerous foci and the overlying dysplastic epithelium (Figure 2).

The comparison between the OSCC and normal oral epithelium did not yield any statistically significant results (Fisher’s exact test, p-value=0.342) as well. Table 3 summarizes the pattern of SOX2 expression.

**Table 3:** Pattern of SOX2 endothelial expression in normal oral epithelium, OL, and OSCC. -: no staining, +: positive staining, ++: more positive staining OL: Oral leukoplakia, OSCC: Oral squamous cell carcinoma

Diagnosis	Positivity
Normal	-
OL (no dysplasia to moderate dysplasia)	+
Severely dysplastic OL and OSCC	++

Findings regarding CD147 expression

**Figure 3:**
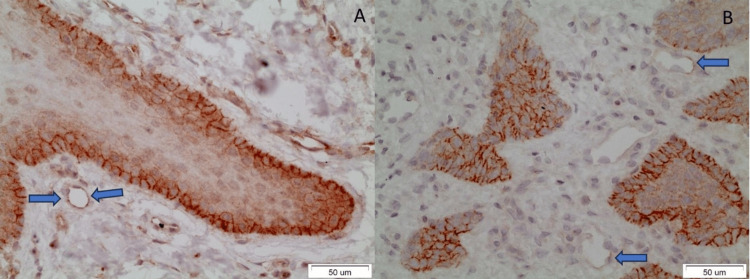
The blue arrows indicate the positively stained endothelial cells. A: Normal oral epithelium (case no. 55), B: Well-differentiated OSCC (case no. 47) OSCC: Oral squamous cell carcinoma

Biomarker CD147 was expressed in the normal oral epithelium, OL epithelium, and OSCC neoplastic cells, but its vascular-endothelial presence was not specific, and it was noticed in only 2/5 (40%) cases of normal oral epithelium, 1/30 (3.3%) cases of OL (one severely dysplastic case), and 4/21 (19%) cases of OSCC (two well-differentiated, one moderately differentiated, and one poorly differentiated case) (Table 4, Figure 3).

**Table 4:** The demographical and clinical manifestation details of the patients from whom the positively stained tissue samples were derived. OL: Oral leukoplakia, OSCC: Oral squamous cell carcinoma

Case no.	Diagnosis	Location	Gender	Age (in years)
8	Severely dysplastic OL	Tongue	Male	43
34	OSCC/Poorly differentiated	Tongue	Female	77
38	OSCC/Moderately differentiated	Tongue	Male	75
47	OSCC/Well-differentiated	Lip	Male	58
48	OSCC/Well-differentiated	Lip	Male	77
52	Normal	Tongue	Female	49
55	Normal	Tongue	Male	69

In the first group, the endothelial cells of vessels were beneath the OL epithelium, displaying cytoplasmic and membranous staining. In cases of OSCC, the positively stained vessels were found at the periphery of the cancer cell islands. The endothelial CD147 staining in OSCC was mild and linear, demarcating the outer wall of the involved vessels. No statistically significant difference was noticed in the OL samples compared to OSCC samples (Fisher’s exact test, p-value=0.146). Furthermore, no statistically significant difference was noticed in the OSCC samples compared to the normal oral epithelium (Fisher’s exact test, p-value=0.146).

## Discussion

Comparative discussion on SOX2

The expression of SOX is detected in higher levels of dysplasia and, in general, in cancer. The positivity increases as dysplasia progresses to higher degrees, whereas in cancer, the positivity isn’t affected by the degree of differentiation. In 56 cases in total, 12 cases were positive, nine of which (75%) corresponded to severely dysplastic or cancerous lesions. It may not be accurately surmised whether this finding originates from neoangiogenesis, typical of cancerous and perhaps precancerous lesions as well, or originates from physiological endothelial expression. It may be the case that this endothelial expression constitutes an early event in tumorigenesis and perhaps displays prognostic value, as premalignant lesions have a higher probability of transitioning to carcinoma. Another possible division corresponds to the localization of vessels; these are in close proximity to the basal membrane of the epithelium and located in deeper layers of the lamina propria. Such observations require larger samples to be statistically proven.

Generally, the SOX transcription factors involved in vascular development include SOX2, SOX7, SOX17, and SOX18 [3,21,35]. The genes SOX7, SOX17, and SOX18 play a significant role in the initial stages of neovascularization, specifically during the commencement of endothelial differentiation and function. These genes are positioned upstream of signaling cascades that control the differentiation process [3,21,35]. The presence of SOX7 haploinsufficiency has been associated with the occurrence of cardiac defects and congenital diaphragmatic hernias [36]. The deletion of the SOX7 gene in mice results in embryonic lethality, which is attributed to the absence of major vessels in the yolk sac and subsequent cardiovascular failure [36]. The loss of SOX17 leads to the depletion of the endoderm and early embryonic lethality [2,37]. The concurrent loss of SOX17 and SOX18 leads to even more severe cardiovascular abnormalities in mice [37]. This implies that there is a degree of functional overlap or redundancy between these two transcription factors. The loss of SOX17 also causes the absence of arteries and the fusion of the aorta with the cardinal vein, which is accompanied by the loss of arteriovenous identity [38]. This observation suggests an underlying correlation between the SOX transcription factors and Notch signaling since the latter plays a key role in the process of arteriovenous differentiation [35]. The Notch signaling pathway has the ability to inhibit the expression of endothelial SOX17 [39]. The transcription factor SOX17 could potentially serve as a mediator in the process of arterial differentiation through Wnt signaling [40]. The loss of SOX18 in mice leads to subcutaneous edema and embryonic lethality as a result of disrupted lymphangiogenesis [41,42]. This observation provides evidence supporting the hypothesis that SOX18 plays a significant role in the process of lymphangiogenesis [41,42]. The transcription factor SOX2 plays a crucial role in the regulation of epithelium-mesenchyme interactions, the differentiation of various cell lineages, and cell fate transitions. It is one of the four primary pluripotent factors, alongside Oct3/4, KLF4, and c-Myc, which are employed in cell reprogramming processes [43] and also function as indicators of neural stem cells [44,45]. Transcription factor SOX2 enhances the ability of cardiovascular cells to undergo reprogramming, induces endothelial differentiation in mesoangioblasts, and contributes to the reprogramming process of corneal endothelial cells. Therefore, SOX2 plays a key role in enhancing the reprogramming capacity of various cell types within the cardiovascular system. The transcription factor SOX2 has been identified as a crucial regulator in the process of neuronal differentiation [46]. This observation implies that both ECs and brain cells share a common origin with progenitor cells, and the process of differentiating into endothelial and neuronal cells is coordinated.

Comparative discussion on CD147

The small number of positive cases and the intensity, as well as the pattern of the staining (mild and linear), do not permit a clear establishment of correlations regarding the CD147 expression in vessels and endothelial cells in normal, premalignant, and malignant tissue samples. Further confusion derived from the fact that the endothelial expression of CD147 was noticed in the limits of the variance: only in a minority of normal and cancerous tissues was endothelial positivity for CD147 observed (absent staining in non-dysplastic, mildly dysplastic, and moderately dysplastic OL). Based on our previous research, CD147 was expected to be observed in normal oral epithelium and OL and OSCC samples [31]. Furthermore, CD147 production is induced in both cancerous and inflammatory microenvironments. Thus, its expression in normal oral epithelium may be attributed to local, proinflammatory agents, or trauma, whereas in cancer, it may be attributed to its potential to regulate and provoke tumorigenesis. The vessels and the endothelial cells are affected as well. The biomarker CD147 induces angiogenesis in human lung carcinomas [47]. The tumor vesicle-associated CD147 regulates the function and properties of endothelial cells, especially their angiogenic potential [48]. Hence, CD147 affects both the phenomena of angiogenesis and metastasis [49]. In skin cancer, i.e., melanoma, it is reported that CD147 plays a significant role in the phenomenon of lymphangiogenesis, which is a precursor to regional lymph node metastasis as well as distant metastasis [27]. In the case of Kaposi's sarcoma-associated herpesvirus, CD147 promotes the tumorigenic potential of the infected endothelial cells [50]. However, CD147's presence may be deemed necessary since its endothelial genetic deletion (such as in the scenario of gene knockout treatments) promotes alterations with regards to the blood-brain barrier, implicating the absence of CD147 in the pathogenesis of Alzheimer’s disease [51].

Limitations

The limitations of our investigation encompassed the absence of patient follow-ups pertaining to the tissue specimens, the absence of tumor-node-metastasis (TNM) categorization, and the absence of information regarding the human papillomavirus (HPV) status of the included OSCCs.

## Conclusions

The presence of SOX2 in the endothelial cells of both potentially malignant and malignant oral lesions indicates that SOX2 may play a role in the formation of new blood vessels and be correlated with the severity of dysplasia in OL. The expression of CD147 can be attributed to both local inflammation and cancer. Expanding the analysis of CD147 to broader cohorts of tissue samples will provide valuable insights into its involvement in cancer and inflammation. The existing evidence justifies the need for further investigations to illustrate the therapeutic relevance of these CSC biomarkers.
